# Social Support, Self-Regulation, and Psychological Skill Use in E-Athletes

**DOI:** 10.3389/fpsyg.2021.722030

**Published:** 2021-11-11

**Authors:** Michael G. Trotter, Tristan J. Coulter, Paul A. Davis, Dylan R. Poulus, Remco Polman

**Affiliations:** ^1^Faculty of Health, School of Exercise and Nutrition Sciences, Queensland University of Technology, Brisbane, QLD, Australia; ^2^Department of Psychology, Umeå University, Umeå, Sweden; ^3^School of Health and Human Sciences, Southern Cross University, Bilinga, QLD, Australia

**Keywords:** esports, video game, competitive video games, cyber sport, e-athlete, self-regulation, social support, psychological skills

## Abstract

The aims of the present study were twofold. First, to investigate self-reported social support, self-regulation, and psychological skill use in esports athletes (e-athletes) compared to traditional athletes. Second, to determine if self-reported social support, self-regulation, and psychological skill use influenced e-athlete in-game rank. An online survey was used to collect data from an international sample of e-athletes (*n* = 1,444). The e-athletes reported less social support, self-regulation, and psychological skill use than reported by traditional athletes in previous studies. E-athletes with higher scores in social support, self-regulation, and psychological skill use was associated with higher in-game rank. The lack of an organisational structure in esports may be a contributing factor as to why e-athletes score lower than traditional athletes on social support, self-regulation, and psychological skill use. Future research is warranted to explore the development of esports programs aiming to promote athletes’ social support, self-regulation, and use of psychological skills to enhance in-game performance and well-being.

## Introduction

Esports has been defined as “organised video game competitions” (Jenny et al., 2017; p. 4) and has shown a rapid growth in terms of its number of e-athletes and spectators over the last 10 years (Newzoo, 2020), and it is currently valued at well over 24 billion dollars (Ahn et al., 2020). The esports industry is noted to have experienced limited disruption as a result of COVID-19 pandemic, with participation rates continuing to rise (López-Cabarcos et al., 2020). There is increasing research activity examining the health and well-being of e-athletes (e.g., Trotter et al., 2020), as well as the psychological factors which potentially influence esports performance (e.g., Himmelstein et al., 2017; Poulus et al., 2020). However, considering the extent of research examining esports, the current understanding of optimal training and coaching methods involved in optimising esports performance is limited.

Based on the definition of esports above, we define e-athletes as those who play an esport and have an official ranking for that esport. Ranking in esports is like the ELO system in chess, however, has different algorithms for each esport (see Poulus et al., 2020). Obtaining a ranking for most esports indicates that the individual has played a minimum number of games and has past the playing for fun stage and now competes to win. There is a need for further research to examine e-athletes’ development to facilitate optimal performance and well-being. Although there are many factors which could potentially influence esports performance (e.g., physical fitness, general health, and stress coping; Polman et al., 2018; Poulus et al., 2020; Trotter et al., 2020), this study focuses specifically on psychological factors (i.e., self-regulation, psychological skill use, and social support). Evidence suggests that social support (Freeman and Rees, 2008; Arnold et al., 2018), self-regulation (Jonker et al., 2011), and psychological skill use (Tod et al., 2011; Brown and Fletcher, 2017; Shaari et al., 2019; Röthlin et al., 2020) can directly or indirectly enhance athletic performance. However, the influence of social support, self-regulation, and psychological skill use on e-athletes’ performance is still relatively unknown, despite previous research suggesting these factors as an area of future research (Freeman and Wohn, 2017; Himmelstein et al., 2017; Brevers et al., 2020).

Previous research into the psychology of e-athletes can be separated into four categories: stress and coping (Smith et al., 2019; Poulus et al., 2020), psychological skill use (Himmelstein et al., 2017), self-regulation (Brevers et al., 2020), and the comparison of e-athletes with traditional sporting athletes (Kang et al., 2020). For example, research has outlined similarities and differences between e-athletes and traditional athletes in terms of stress and coping response and their psychological profiles (Kang et al., 2020; Poulus et al., 2020). In particular, e-athletes report stressors not previously reported by traditional athletes and appraise these stressors as both a challenge and a threat (Poulus et al., in press). Kang et al. (2020) outlined that, compared to baseball players, e-athletes had higher levels of novelty seeking, self-directedness, and self-transcendence, but lower scores in state anxiety.

Research has previously explored psychological skill use (e.g., goal-setting, imagery, and attentional control; Himmelstein et al., 2017), self-regulation (self-directedness, self-efficacy, and self-control; Kang et al., 2020), and received social support (Freeman and Wohn, 2017) in e-athletes. However, to date, no research exists exploring these factors with e-athletes’ in-game rank.

### Social Support

Social support is generally conceptualised as either structural or functional, both of which have been associated with increased sporting performance (Freeman and Rees, 2008). Structural support includes an individual’s network and interconnections of social relationships. Research focusing solely on the exclusive impact of structural support is rare (Freeman, 2020). However, increased structural support has been linked to athletes’ enhanced stress coping and increased performance (Freeman and Rees, 2008; Arnold et al., 2018). Functional support refers to the resources or actions that can come from four dimensions: emotional, esteem, informational, and tangible (Freeman, 2020). Functional support can be categorised as either perceived or received social support (Freeman, 2020). Perceived support relates to the perception of potential access to social support, and received support refers to specific actions of support by an individual’s social networks (Freeman et al., 2011). Evidence shows that received social support positively impacts athletic performance. Freeman and Rees (2008) demonstrated that received social support generated a stress-buffering effect in high-performing golfers. Received social support is also associated with burnout, self-confidence, increased motivation, and performance (Rees et al., 1999; Rees and Freeman, 2007; DeFreese and Smith, 2013; Freeman et al., 2014). To date, there has been no research linking e-athletes’ in-game performances to received social support. However, in a qualitative study, Freeman and Wohn (2019) investigated how esports teams are formed and how they coordinate. In particular, the authors noted that social capital was important in the formation and coordination of effective esports teams. In the formation of esports teams, amateur e-athletes reportedly favoured unstructured recruitment methods (e.g., offline social networks, system matchmaking, and social media crowdsourcing), whereas professional e-athletes preferred formal methods of recruiting teammates. Despite our limited understanding of the role of received social support and how social connections influence esports team formation, no research has explored the link between received functional social support and e-athletes in-game rank. We decided to focus on perceived functional support in the present study because it has been shown to be more consistently related to performance (e.g., Freeman and Rees, 2010), the availability of a reliable and valid tool for its measurement, and to make meaningful comparisons with previous sport studies.

### Self-Regulation

Self-regulation has been described as the “self-generated thoughts, feelings, and actions that are planned and cyclically adapted to the attainment of personal goals” (Zimmerman, 2000, p. 14). Self-regulation involves seven stages, namely: informational input, self-evaluation, instigation to change, search, planning, implementation, and plan evaluation (Brown, 1998; Brown et al., 1999). Self-regulation has been shown to predict performance in sport and academia (Jonker et al., 2011). Jonker et al. (2011) reported that participants with greater self-reported self-regulation scores were likely to be more successful in both academia and elite sports. Other studies have provided further support for this finding, showing that highly self-regulated traditional athletes were more likely to be selected for national level teams in football (Erikstad et al., 2018). Self-regulation skills—such as planning, goal setting, self-monitoring, and reflection—have been described as regular practices used by athletes (Pilgrim et al., 2018).

Self-monitoring and self-reflective skills, associated with the evaluation phase of self-regulation, are noted to be related to higher achievement levels (Jonker et al., 2010; Popa et al., 2020). Traditional athletes, with better self-reflection and self-monitoring skills, are between 2.75 and 11 times more likely to achieve elite level status in sport (Bartulovic et al., 2017; Te Wierike et al., 2018). No empirical research examining the association between self-regulation and esports currently exists. However, Brevers et al. (2020) suggest that similarities between the rule-based nature of esports and traditional competitive sports justifies the investigation into the association between esports and executive functions, such as self-regulation. Further research examining self-regulation is suggested to benefit teachers, athletes, coaches, and parents, who are involved in esports (Brevers et al., 2020).

### Psychological Skill Use

Over the last decade, there has been increasing interest in using sport psychology techniques to maximise competitive sports performance in traditional athletes, through psychological skills training (PST) programs (Meggs and Chen, 2019). PST programs target such skills as goal setting, self-talk, relaxation, imagery, attentional control, emotional control, activation, automaticity, and negative thinking. A reported benefit of psychological skill use is emotional stability (control), which has been shown to account for over 36% of the variance of stress-coping in gymnasts (Woodman et al., 2010). A recent study of the effectiveness of PST programs on 95 athletes, from a variety of sports (e.g., tennis, curling, floorball, badminton), highlighted that increased use of psychological strategies had a positive influence on performance-related factors, such as emotional and attentional control (Röthlin et al., 2020). Similarly, psychological skill use has been found to be employed by e-athletes in training and competition (Himmelstein et al., 2017). In a qualitative study using interviews to explore the psychological skill use of League of Legends e-athletes (*n* = 5) Himmelstein et al. (2017) identified two higher-order themes related to psychological skill use—namely, techniques used to achieve optimal performance and obstacles encountered by competitive gamers. The psychological skills identified in Himmelstein et al.’s (2017) study were related to the higher-order theme optimal performance (e.g., goal setting, imagery, attentional control). The authors suggested that future PST programs would be beneficial for e-athletes. Currently, there are no studies investigating the psychological skill use of e-athletes using existing quantitative measures, and none that compare e-athletes’ use of psychological skills with athletes from traditional sports.

Previous research indicates that social support, self-regulation, and psychological skill use influence traditional sports performance (e.g., Freeman and Rees, 2008; Jonker et al., 2011; Meggs and Chen, 2019). However, there is currently little empirical evidence of these psychological constructs in esports performance research. Therefore, the first aim of this exploratory study was to understand the levels of social support, self-regulation, and psychological skill use that exist in a global esports population and identify how e-athletes compare to traditional athletes on these factors. The second aim was to determine if social support, self-regulation, and psychological skill use are associated with player in-game rank (e.g., achievement level).

## Materials and Methods

### Participants

In total, 2,459 adult e-athletes (742 male, 80 female, 29 other; 1,608 not disclosed) started the study survey, of which 58.7% (*n* = 1,444) completed sufficient and reliable information to be included in the final analysis. E-athletes’ responses were included in the study if their responses were not deemed to be spurious, reported their in-game rank for their respective esport, and completed all items of at least one of the instruments measuring either social support, self-regulation, or psychological skill use.

E-athletes reported the esports games they were most highly ranked in. These games included, Overwatch (*n* = 643), League of Legends (*n* = 410), Counter-Strike Global Offensive (*n* = 193), Rocket League (*n* = 124), and Defence Of The Ancients (DOTA) (*n* = 74). Esports have been categorised into three broad categories, First Person Shooters (FPS), Multiplayer Online Battle Arenas (MOBA) and Sports games. All the games in the present study are MOBA, however in Rocket League e-athletes can achieve an online rank in both solo and team matches. Although the games differ in terms of their mechanics all are similar in that they are games of invasion, where the objective is to enter an opponent’s territory to achieve objectives such as scoring goals or destroying towers.

The participants ranged across all levels of esports experience and were categorised in line with previous research to allow for comparisons across the different esports (see Trotter et al., 2020). The number of e-athletes in each skill rank group was as follows: 0–69% skill group (*n* = 846), 70–79% (*n* = 226), 80–89% (*n* = 175), 90–100% (*n* = 197). In total 843 e-athletes reported their country of residence. In total 65 countries were reported with the majority being from the United States of America (*n* = 290, 34.4%), Australia (*n* = 180 21.4%) Canada (*n* = 75, 8.9%), and Germany (*n* = 41, 4.9%).

### Instruments

All demographic questions were in the English language. Similarly, the original English versions of the instruments below were used.

#### Social Support

Social support was measured using the Athletes Received Support Questionnaire (ARSQ; Freeman et al., 2014). The ARSQ has 22-items and 4 factors: emotional support, esteem support, information support, and tangible support. Emotional support refers to behaviours related to comfort, security, and being cared for (e.g., *In the last week*, *how often did someone cheer you up?*). Esteem support involves activities which support or bolster a person’s self-esteem (e.g., *In the last week*, *how often did someone encourage you?*). Informational support is considered to include advice and guidance (e.g., *In the last week*, *how often did someone give you advice about performing in competitive situations?*). Tangible support is defined as practical and instrumental assistance (e.g., *How often*, *in the last week*, *did someone help plan your training*). The ARSQ is scored on a 5-point Likert scale with 1 = *Not at all*, 2 = *once or twice*, 3 = *three or four times*, 4 = f*ive or six times*, and 5 = *seven or more times*. Freeman et al. (2014) have provided evidence for the factorial structure, convergent validity, and reliability of the ARSQ with athletes competing mainly in British Universities and College sport competitions as well as a sample of competitive athletes across multiple sports. The reliability of the factors of the ARSQ was good for the present study (emotional support α = 0.83; esteem support α = 0.90; informational support α = 0.89; tangible support α = 0.84).

#### Self-Regulation

The Self-Regulation Questionnaire (SRQ; Brown et al., 1999) was used to measure self-regulatory skills. The SRQ consists of 63 items and 6 factors, including evaluating *(e.g., I think a lot about how I*’*m doing)*, triggering *(Usually I see the need to change before others do)*, searching *(There is usually more than one way to accomplish something)*, planning *(Once I have a goal, I can usually plan how to reach it)*, implementing *(I have rules that I stick by no matter what)*, and assessing *(I change the way I do things when I see a problem with how things are going)*. The SRQ is scored on a 5-point Likert scale, ranging from 1 = *strongly disagree* to 5 = *strongly agree*. Scores for individual factors can be calculated, but an overall SRQ score, using all factors, has been recommended (Brown et al., 1999). Scores for an overall self-regulation score can be classified as high (>239), intermediate (214–238), or low (<213; Brown et al., 1999). The SRQ has good reliability (α = 0.91) and validity (*r* = 0.94, *p* < 0.0001) and has been previously used to measure self-regulation in varied sporting populations (Brown et al., 1999; Sadri and Janani, 2015; Shandi et al., 2016; Thapar and Nancy, 2018; Gupta and Sudhesh, 2019). In the present study reliability was moderate to good for five factors and the total scale (receiving α = 0.61; searching α = 0.67; planning α = 0.76; implementing α = 0.73; assessing α = 0.67; total scale α = 0.88) but poor for two factors (evaluating α = 0.44; triggering α = 0.49). Systematic removal of items resulted in removing items 2, 30, and 58 from the evaluation factor and item 59 from the triggering factor (evaluating α = 0.63; triggering α = 0.60).

#### Psychological Skill Use

To measure psychological skill use, the Test of Performance Strategies-2 (TOPS-2; Hardy et al., 2010) questionnaire was used. The TOPS-2 has 64 items, with 36-items related to competition and 36-items related to training. In this study, we only used competition-related items. The TOPS-2 consists of nine factors: goal setting, imagery, relaxation, activation, self-talk, emotional control, automaticity, negative thinking, and distractibility. Distractibility was not included in this study, due to previous research reporting low internal consistency (see Hardy et al., 2010). The TOPS-2 is scored on a 5-point Likert scale ranging from 1 = *strongly disagree* to 5 = *strongly agree*. The TOPS-2 has been shown to have good factorial structure (Hardy et al., 2010). In addition, adequate reliability was reported except for the Distractibility factor. As such this factor was not used in the present study as recommended by Hardy et al. (2010). In the present study Cronbach alpha for the factors of the TOPS-2 were adequate to good (self-talk α = 0.70; emotional control α = 0.81; automaticity α = 0.60; goal-setting α = 0.84; imagery α = 0.79; activation α = 0.72; relaxation α = 0.83; negative thinking α = 0.76).

#### Player Expertise

Esports games categorise e-athletes into skill groups, which indicate player expertise. Skill groups are categorised by in-game rank, which is determined by factors, such as wins and losses and other in-game statistics. The specific in-game statistics, which influence in-game rank, vary from game-to-game and are often not available to the public. In line with previous research (see Poulus et al., 2020; Trotter et al., 2020), this study measured player expertise using player rank.

### Procedure

This study was approved by the institution of the first authors’ research ethics committee (ethics approval number 1800000436). The survey was distributed in two ways: (a) via social media sites, such as Facebook, Twitter, Reddit, and YouTube; and (b) at major esports events in Australia. Prior approval from event organisers was obtained before data collection occurred. Participants who engaged with the social media recruitment strategy were directed to a digital link to the survey, whereas participants collected at esports events completed the survey on an iPad. All participants provided informed consent by agreeing to the survey terms.

### Analysis Strategy

Data were initially screened for outliers and any unrealistic, incomplete, and invalid data were removed leaving 1,444 valid cases. Because of the relatively large sample size, participants who had missing responses for either the ASRQ, SRQ, or TOPS-2 were not included in the analysis for that particular instrument. Normality and homoscedasticity of our dataset was examined. It is well-established that, in large samples (>40), the violation of normality has little influence and parametric statistics can be performed (Ghasemi and Zahediasl, 2012). There was no violation regarding homoscedasticity.

To examine the levels of social support, self-regulation, and psychological skill use in e-athletes, descriptive statistics were computed. In addition, Pearson product moment correlations were calculated between variables, with a small effect <0.30, a medium effect 0.31–0.50, and a large effect >0.51 ranges employed (Cohen, 1988). A one sample *t*-test was then run to compare the mean results of previous studies on social support, self-regulation, and psychological skill use, with the results recorded for the different factors of each scale obtained in the present study. Effect size for the one-sample *t*-test was calculated using Cohen’s d with 0.2 being small, 0.5 medium, and 0.8 and above large (Cohen, 1988).

Multivariate analysis of variance (MANOVA) was conducted to examine differences between social support, self-regulation, and psychological skill use with player rank. In the instance of a significant effect, univariate analysis of variance (ANOVA) was conducted and *post hoc* comparisons were calculated using Sidak. Effect size for the ANOVA was explored using partial eta squared (η*_*p*_*^2^), with a small effect 0.01–0.059, medium effect 0.06–0.139, and a large effect >0.14 (Cohen, 1988).

## Results

Means and standard deviations for the factors associated with social support, self-regulation, and psychological skill use are reported in Table 1. Table 2 provides information on the number of participants in each skill level category, and Table 3 provides information on the number of participants in each self-regulation category, based on their total score.

**TABLE 1:** Means and standard deviation (SD) for received social support, self-regulation, and psychological skills for the sample as a whole and for the different skill categories.

Factor	Mean (SD)	Mean (SD) 0–69%	Mean (SD) 70–79%	Mean (SD) 80–89%	Mean (SD) 90–100%
**Received social support**					

Emotional support	1.99 (0.98)	1.94 (0.94)	2.02 (0.94)	2.11 (0.91)	2.37 (1.02)
Esteem support	1.92 (0.95)	1.84 (0.86)	2.06 (1.02)	2.09 (0.92)	2.31 (1.05)
Information support	1.98 (0.93)	1.91 (0.86)	2.12 (0.95)	2.23 (0.95)	2.40 (1.05)
Tangible support	1.36 (0.60)	1.32 (0.53)	1.38 (0.56)	1.37 (0.58)	1.62 (0.79)

**Self-regulation**					

Informational input	3.66 (0.51)	3.63 (0.52)	3.65 (0.46)	3.75 (0.52)	3.81 (0.49)
Evaluating	3.27 (0.46)	3.27 (0.48)	3.36 (0.42)	3.24 (0.47)	3.33 (0.44)
Triggering	3.48 (0.44)	3.44 (0.42)	3.52 (0.44)	3.56 (0.49)	3.50 (0.45)
Searching	3.76 (0.52)	3.72 (0.50)	3.77 (0.52)	3.74 (0.56)	3.93 (0.49)
Planning	3.20 (0.65)	3.15 (0.63)	3.21 (0.65)	3.25 (0.71)	3.37 (0.65)
Implementing	3.26 (0.64)	3.26 (0.64)	3.24 (0.57)	3.29 (0.66)	3.40 (0.65)
Assessing	3.42 (0.48)	3.39 (0.48)	3.49 (0.43)	3.41 (0.45)	3.51 (0.48)
Total self-regulation	216.45 (22.03	214.68 (21.13)	218.21 (20.48)	218.29 (24.14)	223.59 (21.46)

**Psychological skills**					

Self-talk	2.67 (0.92)	2.61 (0.87)	2.82 (0.97)	2.68 (0.86)	2.95 (0.98)
Emotional control	3.80 (0.89)	3.83 (0.85)	3.69 (0.94)	3.80 (0.92)	3.84 (0.87)
Automaticity	3.41 (0.76)	3.28 (0.72)	3.53 (0.75)	3.53 (0.70)	3.75 (0.74)
Goal setting	2.71 (1.05)	2.57 (1.03)	2.76 (1.05)	2.80 (1.02)	3.04 (1.01)
Imagery	2.64 (1.04)	2.55 (1.03)	2.61 (1.05)	2.56 (0.95)	3.02 (1.03)
Activation	3.01 (0.84)	2.94 (0.81)	3.15 (0.81)	2.99 (0.80)	3.34 (0.78)
Relaxation	2.53 (1.07)	2.52 (1.07)	2.64 (1.06)	2.44 (1.04)	2.60 (1.10)
Negative thinking	2.75 (0.93)	2.74 (0.93)	2.86 (0.95)	2.73 (0.98	2.64 (0.89)

**TABLE 2:** Frequency of esports player in each rank category.

Rank	Frequency	Percent
0–69%	846	58.6
70–79%	226	15.7
80–89%	175	12.1
90–100%	197	13.6
Total	1,444	100

**TABLE 3:** Self-regulation classification based on the total score of the SRQ.

SR classification	Frequency	Percent
Low/impaired	423	42.6
Moderate	427	43.0
High/intact	143	14.4
Total	993	100

Correlational analysis of the study variables is reported in Table 4. Most correlations were positive in nature and all were small to moderate in magnitude. Only the psychological skill, self-talk, was moderately associated with any of the social support factors. In addition, there were several moderate associations between the psychological skill use and self-regulatory factors. For example, both searching and assessing was moderately associated with self-talk, goal setting, imagery, and activation; whereas, planning and implementing were moderately positively associated with emotional control and activation, and moderately negatively correlated with negative thinking.

**TABLE 4:** Pearson product moment correlations between study variables.

	Received social support (ARSQ)				Self-regulation (SRQ)						
Variable	Emotional support	Esteem support	Info support	Tangible support	Informational input	Evaluating	Triggering	Searching	Planning	Implementing	Assessing
**Psychological skills (TOPS-2)**											

Self-talk	0.30	0.35	0.31	0.32	0.21	0.13	0.17	0.37	0.22	0.22	0.30
Emotional control	–0.08	–0.08	–0.13	–0.16	0.20	–0.23	0.20	0.17	0.35	0.38	0.08
Automaticity	0.11	0.15	0.13	0.11	0.14	0.01	0.06	0.22	0.18	0.26	0.13
Goalsetting	0.21	0.26	0.26	0.27	0.29	0.20	0.18	0.36	0.33	0.22	0.45
Imagery	0.26	0.29	0.26	0.27	0.17	0.15	0.12	0.30	0.20	0.12	0.29
Activation	0.22	0.28	0.27	0.28	0.27	0.04	0.23	0.43	0.38	0.37	0.33
Relaxation	0.18	0.25	0.18	0.25	0.13	0.15	0.14	0.29	0.08	0.11	0.27
Negative thinking	–0.02	–0.06	0.02	0.00	–0.18	0.22	–0.21	–0.24	–0.38	–0.33	–0.14

**Received social support (ARSQ)**											

Emotional support					0.05	0.10	0.04	0.22	0.07	0.10	0.12
Esteem support					0.12	0.11	0.07	0.26	0.09	0.11	0.17
Informational support					0.10	0.14	0.04	0.21	0.05	0.0	0.16
Tangible support					0.08	0.09	–0.03	0.17	0.04	0.06	0.14

To address our first aim, we compared e-athletes scores on the ARSQ, TOPS-2, and SRQ with studies on traditional athletes which used the same measures. To date, six studies have used the ARSQ. We compared our data with that reported by Hartley and Coffee (2019), because they used the English version of this instrument and collected data from athletes from multiple sports (*n* = 54) with a mean age of 26 years and mixed achievement levels (from recreational to international). Of the four studies that have used the SRQ, we used a study, conducted by Sadri and Janani (2015), on 100 male swimmers with a mean age of 23 and 11 years of experience on average, as it is the only study to report scores for the individual SRQ factors. Finally, for the TOPS-2, we compared our findings with those of Kruk et al. (2017), with male soccer players aged between 16 and 27 years (*M* = 18.2 years) from regional clubs with 3–15 years playing experience (*M* = 9.33 years), because it is one of only two studies which used the competition version of the TOPS-2. The other study, meeting this criterion, was deemed unsuitable, because the participants were army recruits (Fitzwater et al., 2018).

Overall, we compared the findings of the present study with results obtained in a sample of 222 athletes, ranging from recreational to international competition levels in cycling, rugby, and soccer (Hartley and Coffee, 2019; see Table 5). This comparison showed that the e-athletes reported significantly lower levels of esteem, emotional and tangible support, but similar levels of informational support. For self-regulation, e-athletes scored significantly lower for evaluating, searching, planning, implementing, assessing, and total self-regulation, but higher for triggering. Finally, for psychological skill use, e-athletes reported significantly less use of self-talk, automaticity, goal setting, imagery, relaxation, negative thinking and activation, but higher use of emotional control.

**TABLE 5:** *T*-test comparisons between traditional sports and esports data (**P < 0.001).

Factor	N	Mean (SD)	Comparison M	t	sig	Mean difference	Cohen’s d	CI
**Received social support**								

Emotional support	1,222	1.99 (0.98)	2.16	–6.02	>0.001**	–0.22		−0.22/−0.11
Esteem support	1,222	1.92 (0.95)	2.41	–17.86	>0.001**	–0.43		−0.54/−0.43
Informational support	1,222	1.98 (0.94)	1.96	0.982	0.33	0.02		−0.02/−0.08
Tangible support	1,222	1.37 (0.60)	1.82	–26.15	>0.001**	–0.45		−0.48/−0.42

**Self-regulation**								

Informational input	993	3.66 (0.51)	3.68	–1.30	0.19	–0.02	–0.04	−0.05/0.01
Evaluating	993	3.27 (0.46)	3.61	–22.79	>0.001**	–0.34	–0.72	−0.36/−0.31
Triggering	993	3.48 (0.44)	3.21	18.92	>0.001**	0.27	0.60	0.24/0.29
Searching	993	3.76 (0.52)	4.04	–17.31	>0.001**	0.28	–0.55	−0.32/−0.25
Planning	993	3.20 (0.65)	3.47	–12.92	>0.001**	0.27	–0.41	−0.31/−0.23
Implementing	993	3.26 (0.64)	3.69	–21.02	>0.001**	–0.42	–0.67	−0.47/−0.39
Assessing	993	3.42 (0.48)	3.67	–16.47	>0.001**	–0.25	–0.52	−0.28/−0.22
Total self-regulation	993	216.45 (22.02)	228.56	–17.32	>0.001**	–12.11	–0.55	13.48/10.73

**Psychological skills**								

Self-talk	838	2.67 (0.92)	3.33	–20.67	>0.001**	–0.66		−0.72/−0.60
Emotional control	838	3.80 (0.89)	2.31	48.21	>0.001**	1.49		1.43/1.55
Automaticity	838	3.41 (0.76)	3.64	–8.63	>0.001**	–0.23		−0.28/−0.18
Goal setting	838	2.71 (1.04)	3.91	–33.28	>0.001**	–1.20		−1.27/−1.13
Imagery	838	2.64 (1.04)	3.71	–29.95	>0.001**	–1.07		−0.15/−1.00
Activation	838	3.01 (0.84)	3.94	–32.18	>0.001**	–0.93		−0.99/−0.88
Relaxation	838	2.53 (1.07)	2.92	–10.55	>0.001**	–0.39		−0.46/−0.32
Negative thinking	838	2.75 (0.93)	3.01	–8.16	>0.001**	–0.26		−0.33/−0.20

The MANOVA exploring the association between player rank and social support was significant (Wilk’s lambda = 0.94, *p* < 0.001; η*_*p*_*^2^ = 0.02). Follow up ANOVAs showed significant differences for esteem support [*F*(3, 1,000) = 11.81, *p* < 0.001; η*_*p*_*^2^ = 0.034], emotional support [*F*(3, 1,000) = 8.80, *p* < 0.001; η*_*p*_*^2^ = 0.03], informational support [*F*(3, 1,000) = 13.95, *p* < 0.001; η*_*p*_*^2^ = 0.03], and tangible support [*F*(3, 1,000) = 10.80, *p* < 0.001; η*_*p*_*^2^ = 0.031] all with a small effect size. *Post hoc* comparisons showed that the top 10% skill group of e-athletes received more emotional and information support than those ranked 79% or below, and more esteem support than those ranked in the bottom 69%. Similarly, those ranked 80–89% received more esteem and informational support than those ranked 69% or below.

The MANOVA for self-regulation and player rank was significant (Wilk’s lambda = 0.94, *p* < 0.001; η*_*p*_*^2^ = 0.21). Follow up ANOVAs showed significant differences for input [*F*(3, 818) = 5.45, *p* = 0.001; η*_*p*_*^2^ = 0.02], triggering [*F*(3, 818) = 2.69, *p* = 0.045; η*_*p*_*^2^ = 0.01], searching [*F*(3, 818) = 5.85 *p* = 0.001; η*_*p*_*^2^ = 0.02], planning [*F*(3, 818) = 4.22, *p* = 0.01; η*_*p*_*^2^ = 0.02], and assessing [*F*(3, 818) = 3.33, *p* = 0.02; η*_*p*_*^2^ = 0.012] all with a small effect size. No significant differences were found for evaluating [*F*(3, 818) = 0.47, *p* = 0.09; η*_*p*_*^2^ = 0.01] or implementing [*F*(3, 818) = 0.84, *p* = 0.10; η*_*p*_*^2^ = 0.01]. *Post hoc* comparisons showed that the top 10% skill group of e-athletes reported significantly higher scores for informational input, searching, planning, and assessing behaviour than the 0–69% group and, for searching, higher than the 80–89% group. No significant differences were found for triggering.

The MANOVA for psychological skill use and player rank was significant (Wilk’s lambda = 0.89, *p* < 0.001; η*_*p*_*^2^ = 0.37). Follow up ANOVAs showed significant differences for self-talk [*F*(3, 697) = 4.76, *p* = 0.003; η*_*p*_*^2^ = 0.02], automaticity [*F*(3, 697) = 14.90, *p* < 0.001; η*_*p*_*^2^ = 0.06], goal-setting [*F*(3, 697) = 6.73, *p* < 0.001; η*_*p*_*^2^ = 0.03], imagery [*F*(3, 679) = 6.80, *p* < 0.001; η*_*p*_*^2^ = 0.03], and activation [*F*(3, 679) = 8.50, *p* < 0.001; η*_*p*_*^2^ = 0.04] all with a small effect size except for automaticity which had a medium effect size. No significant differences were found for emotional control [*F*(3, 696) = 0.75, *p* = 0.75; η*_*p*_*^2^ = 0.003], relaxation [*F*(3, 696) = 0.76, *p* = 0.52; η*_*p*_*^2^ = 0.003], or negative thinking [*F*(3, 696) = 0.76, *p* = 0.39; η*_*p*_*^2^ = 0.004]. *Post hoc* comparisons showed that the e-athletes in the top 10% skill group scored significantly higher on positive self-talk and automaticity, compared to the 0–69% skill group, and imagery and activation with all other skill groups. For automaticity, the 0–69% skill group scored significantly lower than all other skill groups.

## Discussion

This study examined the level of social support, self-regulation and psychological skill use in e-athletes in comparison to traditional athletes and how this was associated by in game rank (achievement level). Findings showed that e-athletes received less social support, reported less self-regulations and generally had a lower level of psychological skill use compared to traditional athletes. However, this was influenced by skill level with those in the top 10% reporting higher levels of social support, self-regulation and some psychological skill use.

In this study, e-athletes reported less received social support than traditional athletes. Specifically, e-athletes received less esteem, emotional, and tangible social support, but similar levels of informational social support, compared to traditional athletes. The similar levels of received support, compared to traditional athletes, may indicate that informational support is one of the most influential forms of received social support in esports, and thus e-athletes report its use more frequently. Previous research has suggested that informational support and tangible support are the foundations for esteem and emotional support in an esports context. Specifically, Freeman and Wohn (2017) suggested that most e-athletes started out as strangers and that in-game acts of helping were in the form of informational and tangible support. One way that informational support can manifest is in the form of in-game “pings,” which provide e-athletes with a quick targeted method of communication to increase situational awareness (Leavitt et al., 2016). As e-athletes begin to compete together more regularly, and their relationships become more complex, informational and tangible support lead to more emotional and esteem support (Freeman and Wohn, 2017). Despite the potential for functional social support to be developed through in-game relationships, other avenues for support, such as through a coach, are less likely in esports, compared to traditional sports, due to the lower number of esports developmental or grassroots programs. In traditional sport, social support has been associated with improved performance (Rees and Freeman, 2007; Freeman et al., 2009) and well-being (Holt and Hoar, 2006). It would be important to explore how e-athletes reporting lower levels of social support can enhance their levels of received functional social support. Hence, increasing levels of received functional social support could be beneficial by acting as stress-buffering (Rees and Freeman, 2007).

In this study, it was found that e-athletes had low-to-moderate levels of self-regulation and significantly lower total scores of self-regulation when compared to traditional athletes. Additionally, e-athletes also reported significantly lower self-regulatory scores for evaluation, searching, planning, implementing, and assessing factors compared to traditional athletes. However, they had higher scores for triggering. It is theorised that the lower scores of self-regulation in e-athletes stems from the lack of access to esports coaching. Self-regulation has been shown to be developed through the co-regulation of self-regulatory skills by coaches and parents (Collins and Durand-Bush, 2014). The low-to-moderate levels of self-reported self-regulation by e-athletes, and significantly lower levels of self-regulation compared to traditional athletes, might be explained by the lower number of developmental and grassroots esports programs compared to traditional sport. As esports developmental and grassroots programs are not as common as traditional sports programs, there may not be as much opportunity for e-athletes to be exposed to relationships who could co-regulate and develop self-regulatory skills.

Triggering refers to conscious use of internal or external cues to correct behaviour which is not working. Esports have set parameters or rules. E-athletes are likely to get attuned to the rules, constantly scanning for information to make changes to gain an advantage or maintain dominance over their opponents. As esports is played in the digital world these cues are likely to be more consistent and recognisable compared to the physical world. As such triggering might be developed more in e-athletes compared to traditional athletes.

Finally, all self-reported psychological skills used by e-athletes differed significantly from the psychological skill use reported by traditional athletes. Results indicated that e-athletes reported significantly less use of self-talk, automaticity, goal setting, imagery, activation, relaxation, and negative thinking than swimmers (Kruk et al., 2017). However, e-athletes reported significantly greater use of emotional control. Previous, qualitative research, with a small elite sample of League of Legends athletes, has shown that they reported the use of goal setting, flow (automaticity), and emotional self-regulation to enhance their performance (Himmelstein et al., 2017; Poulus et al., in press). Previous research suggests that PST programs for traditional athletes are effective for increasing athlete psychological, behavioural and performance outcomes (Barker et al., 2020). It is likely that most of the e-athletes in the present study have not been exposed to strategies to develop their psychological skills (e.g., through coaching or psychological support), despite previous research indicating that e-athletes would benefit from such training (Himmelstein et al., 2017).

Interestingly, emotional control was found to be higher in e-athletes compared to traditional sports athletes but did not vary based on player rank. It is possible that emotional control is used more by e-athletes than the comparison group of traditional sports, due to the high level of anti-social behaviour reported by e-athletes. Poulus et al. (in press) reported that anti-social behaviour was the highest reported, second-order stressor and represents a significant source of stress for competitive e-athletes. It is possible that exposure to anti-social behaviour in esports games has led to development of increased emotional control as a coping mechanism across all e-athletes. Further research is required to better understand how emotional control is developed and used by e-athletes.

Considering the notion that higher levels of received social support have been associated with increased performance (Rees and Freeman, 2007; Freeman et al., 2009), it is not surprising that the e-athletes ranked in the top 10% reported higher levels of social support across the four factors assessed. The cross-sectional nature of the study does not allow us to say whether increased social support resulted in higher ranking or vice versa. However, the notion that social support is considered to be important for the well-being of athletes (e.g., Rees and Hardy, 2000) suggests that future research could examine whether this is the case for e-athletes. Secondly, it would be important to examine whether higher levels of received social support are related to the different organisational structures in esports. As indicated, higher ranked e-athletes are more likely to play in established teams with coaches and/or other support staff providing greater opportunities for social support, compared to those who compete online alone.

As indicated, previously, self-regulatory skills have been shown to be related to higher levels of sport performance (e.g., Jonker et al., 2011). Importantly, all of the stages of the cyclical process of self-regulation, as defined by Zimmerman (2000), have been associated with increased performance in traditional sports (Toering et al., 2009; Jonker et al., 2010; Popa et al., 2020). The results from this study indicate that, just as in traditional sports, being ranked higher as an e-athlete was associated with more refined self-regulatory skills. In particular, skills associated with forethought and self-reflection phases of self-regulation were significantly associated with higher rank in e-athletes. This finding is likely because e-athletes, who invest time to increase their skills by selecting strategies and goals directed at increasing their skills, and then reflect on the effectiveness of those strategies and goals, are more likely to increase their performance.

Higher player rank was also associated with greater use of the following psychological skills: self-talk, automaticity, imagery, and activation. These psychological skills have been associated with higher levels of sports performance (e.g., Cheng et al., 2015; Arthur et al., 2017; Simonsmeier and Buecker, 2017). Importantly, self-talk, goal setting, and imagery are also associated with Zimmerman’s (2000) cyclical theory of self-regulation. As such, the higher levels of self-regulation reported by the top 10% of e-athletes might be partially due to greater use of some psychological skills.

Despite the generally low scores, the positive and significant relationships social support, self-regulation, and psychological skill use have with esports player rank is encouraging. In particular, because these factors can be developed e-athletes wishing to increase their ranking may seek out opportunities to take part in developmental programs targeting these aspects of esports high performance.

Despite best efforts, this study has some limitations. Firstly, participant responses could have been impacted by the self-report measures used. For example, it is possible that participants may have answered the surveys in a way they believed was socially desirable. Second, this study was only distributed in English, which may have impacted the number of participants who were able to take part from non-English speaking countries. Thirdly, the study was cross-sectional in nature which only allows for associations and not causation. Fourth, the e-athletes in this study competed in MOBA esports. The traditional athletes in the comparison studies for social support and psychological skill use competed in diverse but mainly team sports. However, for self-regulation the comparison sample consisted of swimmers. To date it is unclear whether participating in a team or individual sport result in varied development of self-regulatory skills. A final limitation of this study is the single cohort nature of the comparison between e-athletes and traditional athletes. In support of previous research (Himmelstein et al., 2017; Brevers et al., 2020), we also recommend that future studies investigate interventions focused on the development of social support, self-regulation, and psychological skill development through developmental esports programs, such as youth grassroots esports competitions in high-schools or the general community. Future research could also investigate the long-term effects of developmental and grassroots esports programs and competitions.

The present study’s findings highlight that social support, self-regulation, and the use of psychological skills in e-athletes are generally lower than those observed in traditional sporting populations. Further, it is noted that social support, self-regulation, and psychological skill use are significantly and positively related to esports player rank. In summary, developmental esports programs may have the potential to promote effective and generalisable self-regulation skills. In comparison with traditional sport development programs, social support (e.g., coaches, parents) is an underused resource that can potentially foster the co-regulation of psychological skill use to increase in-game performance and future life outcomes.

## Data Availability Statement

The raw data supporting the conclusions of this article will be made available by the authors, without undue reservation.

## Ethics Statement

The studies involving human participants were reviewed and approved by the Queensland University of Technology - Office of Research Ethics and Integrity, Research Ethics Advisory Team. The patients/participants provided their written informed consent to participate in this study.

## Author Contributions

MT and RP: conceptualisation, formal analysis, data curation, and project administration. MT, RP, and DP: methodology and investigation. RP: resources. MT: writing—original draft preparation. MT, TC, PD, DP, and RP: writing—review and editing. TC, PD, and RP: supervision. All authors have read and agreed to the published version of the manuscript.

## Conflict of Interest

The authors declare that the research was conducted in the absence of any commercial or financial relationships that could be construed as a potential conflict of interest.

## Publisher’s Note

All claims expressed in this article are solely those of the authors and do not necessarily represent those of their affiliated organizations, or those of the publisher, the editors and the reviewers. Any product that may be evaluated in this article, or claim that may be made by its manufacturer, is not guaranteed or endorsed by the publisher.
